# Mitochondrial Dysfunction and Oxidative Stress: Emerging Insights in Muscle and Cardiovascular Disease Mechanisms

**DOI:** 10.3390/antiox14080902

**Published:** 2025-07-23

**Authors:** Marianne Riou, Bernard Geny

**Affiliations:** 1 Team 3072 “Mitochondria, Oxidative Stress and Muscle Plasticity”, CRBS, Translational Medicine Federation of Strasbourg (FMTS), University of Strasbourg, 1 Rue Eugène Boeckel, CS 60026, 67084 Strasbourg, France; 2 Physiology and Functional Exploration Service, University Hospital of Strasbourg, 1 Place de L’hôpital, 67091 Strasbourg, France

Beyond their role as the “energy powerhouse” of the cell, mitochondria have emerged as essential actors in molecular signaling and determination of cellular fate, particularly through the production of reactive oxygen species (ROS). While traditionally considered as an unregulated process at the origin of oxidative stress and pathology, growing evidence demonstrates that ROS are also signaling molecules, enabling numerous physiological processes.

As the primary source of intracellular ROS, the mitochondrial electron transport chain (ETC) has attracted significant interest. Thus, investigating mitochondrial respiration provides valuable insights into mitochondrial dysfunction in tissues or cells. Indeed, any abnormality in the process of substrate oxidation or in components of the oxidative phosphorylation (OXPHOS) system may lead to a reduction in high-energy phosphate production, resulting in tissue dysfunction, systemic disease and potentially death [1,2].

Given their high mitochondrial content, cardiac and skeletal muscles represent ideal tissues for studying mitochondrial dysfunction [3,4,5,6,7]. Moreover, growing evidence suggests that therapies designed to reduce mitochondrial dysfunction may represent promising therapeutic strategies for muscular and cardiovascular diseases. Consequently, there is a challenge to develop specific and safe metabolic and antioxidant interventions—either alone or in combination—that target the mitochondrial OXPHOS system to improve heart failure therapy [8,9,10].

In this way, five papers were published in the Special Issue of *Antioxidants*, entitled “Mitochondrial functions and oxidative stress during cardiorespiratory and muscular diseases” [11,12,13,14,15]. These contributions highlight novel mechanisms and contribute to the current understanding of mitochondrial dysfunction in skeletal muscle, left heart failure and pulmonary hypertension (PH). They also underscore the role of oxidative stress and identify algae as a promising source of antioxidants.

Chernyavskij et al. investigated myogenesis in human myoblasts. Using various markers and oxidative stress modulators, the authors observed that the suppression of myogenesis by the tumor necrosis factor was associated with a mitoROS-dependent increase in general autophagy and mitophagy [11]. Considering that heart failure is a systemic condition associated with muscle damage [16,17,18,19], these findings suggest a shared mechanism of mitochondrial dysfunction in both cardiovascular and muscular diseases.

Zanini et al. explored the role of the mitochondrial protease Lonp1 in the regulation of mitochondrial function in cardiovascular and muscular diseases [12]. Lonp1 modulates multiple mitochondrial functions in both cardiac and skeletal muscles. Notably, Lonp1 knockout stops heart development and induces cardiomyocyte apoptosis. Similarly, the specific ablation of Lonp1 in mice results in reduced skeletal muscle fiber size and strength.

In addition to the involvement of Lonp1 in heart development, Bhullar et al. focused on the role of substrate oxidation and ETC impairments, resulting in the depletion of myocardial high-energy phosphates, mitochondrial calcium overload and increased levels of ROS in the failing heart [13]. Accordingly, interventions improving ATP production have been reported to be a beneficial therapeutic strategy for heart failure.

By comparing with the physiopathology of the left heart, right heart failure resulting from PH is also associated with mitochondrial dysfunction in muscle tissues [20,21,22,23,24]. Ryanto et al. reviewed the large contribution of mitochondrial dysfunction to PH pathogenesis at different levels, including metabolism and the pulmonary vessels [14]. They also proposed that targeting mitochondrial metabolic pathways may provide new therapeutic issues [14,25].

Finally, pursuing an original therapeutic approach, Vignaud et al. described the antioxidant potential of microalgae in modulating oxidative stress in skeletal muscle [15]. Although they require additional investigation, microalgae and their biomolecules may reduce ROS-induced muscle alterations, especially in conditions such as exercise-induced stress or muscular diseases. This approach is particularly relevant in the context of environmental pollutants such as microplastics, which have been shown to induce skeletal muscle insulin resistance through mitochondrial dysfunction and ROS overproduction [26].

Taken together, these data address several knowledge gaps and provide new insights for improving mitochondrial function in muscle and cardiovascular diseases. Another emerging approach is using mitochondrial transplantation to reduce tissue mitochondrial dysfunction. Transplanting viable and functional mitochondria from non-ischemic tissues into injured myocardium is a promising approach for replacing dysfunctional mitochondria, promoting tissue repair and treating ischemic heart disease. Such an approach is potentially useful in almost all pathologies, including muscular and cardiopulmonary conditions, although the identification of a suitable source of mitochondria and precise techniques are still under investigation [27,28,29,30,31].

Moreover, although peripheral muscle biopsies are relatively easy to obtain in humans, they remain invasive. On the other hand, cardiac biopsies are risky and limited to the setting of cardiac surgery. A less invasive alternative is to study mitochondrial function in circulating cells, such as peripheral blood mononuclear cells [PBMCs] or platelets, which is also an area of increasing interest. Several publications have reported a relationship between the mitochondrial respiration of circulating cells and the severity of heart and lung diseases. Furthermore, as these cells have specific characteristics, such as inflammatory and immune functions in the case of PBMCs, studying their mitochondrial respiration and ability to produce ROS could improve the diagnosis, prognosis and potentially the therapy of many systemic diseases in which mitochondrial respiration is impaired [32,33,34,35,36,37,38,39,40,41].

In conclusion, there are many promising opportunities to improve our understanding of and develop targeted therapies for mitochondrial dysfunction and oxidative stress in muscle and cardiovascular diseases. Active research in this area may likely offer the prospect of repairing skeletal muscles and “broken hearts” in the future.
